# Comparing the anti-bacterial performance of chlorination and electrolysis post-treatments in a hand washing water recycling system

**DOI:** 10.1016/j.wroa.2018.100020

**Published:** 2018-12-14

**Authors:** Christopher Ziemba, Odile Larivé, Svenja Deck, Theo Huisman, Eberhard Morgenroth

**Affiliations:** a Eawag: Swiss Federal Institute of Aquatic Science and Technology, 8600, Dübendorf, Switzerland; b ETH Zürich, Institute of Environmental Engineering, 8093, Zürich, Switzerland

**Keywords:** Greywater, Regrowth, Decentralized, Assimilable organic carbon (AOC), Biologically activated membrane bioreactor (BAMBi), Hydroxyl radical

## Abstract

Innovative solutions are necessary to enable the decentralized recycling of greywater for applications requiring high-quality water, such as hand washing. While physical barriers such as ultrafiltration membranes effectively prevent the passage of bacteria, and chemical and biological treatments can effectively reduce the carbon content of the treated water, there exists a knowledge gap regarding the application of anti-bacterial strategies to prevent the growth of harmful bacteria following treatment. In this study, the effluent water from a household-scale greywater treatment system was fed to seven parallel experimental post-treatment tanks: three receiving direct chlorination with free chlorine residuals of 0.2, 1 or 5 mg Cl_2_/L, three with chlorine produced through electrolysis at the same residual concentrations, and one control with no chlorine added. For increasing concentrations of direct chlorination, the median total cell count (TCC) values were 9 × 10^4^, 2.9 × 10^4^ and 1.8 × 10^3^ cells/mL, respectively. Electrolysis treatment produced very similar TCC concentrations, 8.8 × 10^4^, 1.1 × 10^4^ and 2.3 × 10^3^ cells/mL. The TCC concentrations were lower than the concentration of the water entering each tank (∼3 × 10^5^ cells/mL). Intact cell count (ICC) measurements indicated that the viable cell concentrations, were less than 10% of the TCC values. Though electrolysis treatment can produce powerful oxidants, such as hydroxyl radical, there was no evidence that electrolysis in this system provided additional benefits beyond chlorine production for control of total or intact cell counts. Oxidation of bacteria by chlorine was the dominant anti-bacterial mechanism in our system. Monitoring of dissolved organic carbon (DOC) and assimilable organic carbon (AOC) did not suggest that carbon-limitation significantly impacted cell counts when chlorination or electrolysis treatment was applied. This work demonstrates that either direct chlorination or electrolysis treatment are able to reduce bacteria concentrations over long-term operation of a hand washing water treatment system. We recommend selecting chlorine residual targets such that a chlorine residual is maintained during periods of challenging operating conditions. We observed that a target residual of 1 mg Cl_2_/L, in our system, maintained the TCC below the concentration found in Zürich drinking water.

## Highlights

• Electrolysis and chlorination cell counts were below drinking water concentrations.
• Electrolysis treatment did not yield lower cell counts than chlorination.
• Inactivation attributed to oxidation damage of cell membranes and nucleic material.
• Chlorine residual targets should provide residual during disruptive operation.
• We recommend a free chlorine residual target of 1 mg/L in our system.

## Graphical abstract

**Figure:**
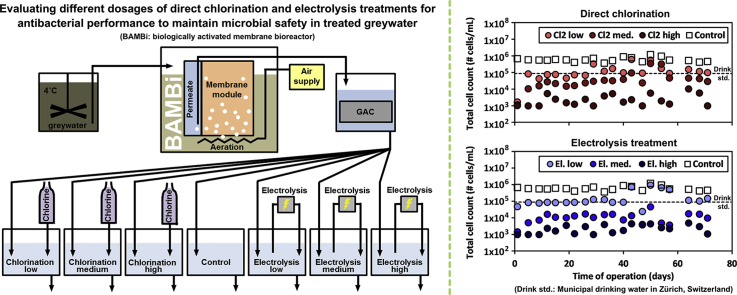

## 1 Introduction

The Sustainable Development Goals (SDGs) Target 6.2 calls for “access to adequate and equitable sanitation and hygiene for all” by 2030 (UN, 2015). Yet, substantial progress is needed to meet this goal: an estimated 47% of the population in the least developed countries lacked access to any hand washing facilities, and a further 27% had access to hand washing facilities without soap or water (WHO and UNICEF, 2017). The SDGs promote hand washing in part because of the potential impacts on human health: washing hands with soap could reduce the risk of diarrheal diseases by 42–47% (Curtis and Cairncross, 2003). Enabling hand washing by constructing centralized water treatment and distribution networks is not practical in many regions of the developing world, due to high costs of installation and maintenance, uncertainties linked to population growth and institutional instability (Larsen et al., 2016). Household-scale systems that recycle hand washing water may offer a more practical solution to improve sanitation in these areas, but only if these systems can be used safely. Many existing greywater treatment systems utilize a disinfection step to reduce bacteria concentration at the effluent. The most common form of disinfection in greywater treatment is chlorination (Boyjoo et al., 2013), but in no previous case has the treatment system been designed to provide hand washing quality water and the chlorination configured to meet this objective. There exists a specific knowledge gap in our understanding of how to configure a modern greywater treatment system with an effective chlorine disinfection step to achieve a water quality sufficient for hand washing. This study seeks to close this gap by quantifying the anti-bacterial performance and identifying the mechanisms of anti-bacterial activity in water produced by an effective greywater treatment system, subject to different chlorination strategies. This study utilizes a previously developed water treatment system known as a biologically activated membrane bioreactor (BAMBi) (Künzle et al., 2015). Our primary reasons for selecting the BAMBi system are, (i) the simplicity, small footprint and the low-maintenance operation of the BAMBi make the system practical and affordable for lower-income communities (Künzle et al., 2015; Ravndal et al., 2015), (ii) long-term testing has demonstrated stable operation and effective contaminant removal from synthetic hand washing water (Ziemba et al., 2018). The core technology of the BAMBi is an ultrafiltration membrane submerged in a treatment tank. Aeration below the membrane promotes the development of a thick but permeable biofilm on the membrane surface (Künzle et al., 2015). Long-term testing with a BAMBi fed a mixture of feces, urine, soap and water has demonstrated 95% removal of organic carbon (Künzle et al., 2015) as well as stable nitrification and denitrification (Ravndal et al., 2015). Similar performance has been demonstrated treating a synthetic hand washing greywater (Ziemba et al., 2018). BAMBi utilizes a gravity-driven membrane (GDM) which requires no additional pressure to be applied to the feed side of the membrane beyond the weight of water. The flux of water through the membrane is lower than would be observed in a conventional membrane bioreactor (MBR), however, the biofilm that develops in the GDM system is self-regulating and requires no fouling control during operation (Derlon et al., 2012; Peter-Varbanets et al., 2010).

The ultrafiltration membrane in the BAMBi presents a physical barrier to the passage of pathogens to the treated water. Testing in our laboratory has demonstrated removal of 7-log bacteria and 3-log MS-2 bacteriophage (unpublished data). A significant concern however, is the growth of bacteria that may be able to enter into the water after the membrane, while the water is stored until use. Pathogen growth testing has demonstrated that the permeate of a BAMBi system, treating a wastewater similar in composition to greywater, can still contain significant concentrations of assimilable organic carbon (AOC), dissolved organic carbon (DOC) and other nutrients, sufficient to support the growth of *Escherichia coli* and *Pseudomonas aeruginosa* (Nguyen et al., 2017). Therefore some form of disinfection or post-treatment is necessary to maintain the safety of the water (Nguyen et al., 2017).

Free chlorine (HOCl) is a strong oxidant, which affects a cell in many ways, including damaging membranes, disrupting membrane functions and damaging DNA. Batch testing with permeate water from a BAMBi system treating hand washing water mixed with flushing water from a source-separating toilet, demonstrated that a free chlorine concentration of 0.5 mg/L could inactivate 5-log of spiked *Escherichia coli* within 5 min of exposure (Nguyen et al., 2017). March et al. (2004) showed total inactivation of coliforms with a 1 mg/L free chlorine concentration in greywater following treatment with a 0.3 mm filter and sedimentation tank. The amount of free chlorine that needs to be added to a system to reach a target concentration must first overcome the chlorine demand, which is the amount of added chlorine that will rapidly react with organics and other compounds, such as ammonium and nitrite (Lazarova et al., 1999; Winward et al., 2008). Chlorine can either be added from a reservoir, or produced using electrolysis. Electrolysis can also produce other reactive oxidants such as hydroxyl radicals, with greater oxidation potential than chlorine (Parsons and Williams, 2004), which may more effectively inactivate bacteria by direct oxidation (Jeong et al., 2006), or may more-effectively oxidize organic materials in the water (Kraft, 2007; Martínez-Huitle et al., 2015), which could otherwise be used as substrate by the bacteria (AOC and DOC). Therefore, a treatment system utilizing electrolysis to produce a certain concentration of chlorine, may result in lower bacteria concentrations than a system in which the same concentration of chlorine is added directly. In the context of a decentralized system, electrolysis may offer an opportunity to produce sufficient chlorine without requiring refilling of a chemical chlorine dosing reservoir.

The specific goals of this study were to (i) evaluate bacterial content in systems employing different dosages of direct chlorination and electrolysis treatment, (ii) compare the anti-bacterial mechanisms employed in each condition (direct inactivation of bacteria, direct growth inhibition or indirect growth inhibition by carbon-limitation), and (iii) recommend optimal post-treatment solutions for systems treating hand washing water or similar water types such as rainwater or greywater from other sources. Understanding the performance and mechanisms for each post-treatment, as well as how this performance could be affected by challenging operating conditions were ultimately necessary to determine an optimal process configuration.

## 2 Materials and methods

### 2.1 System overview

**Fig. 1:**
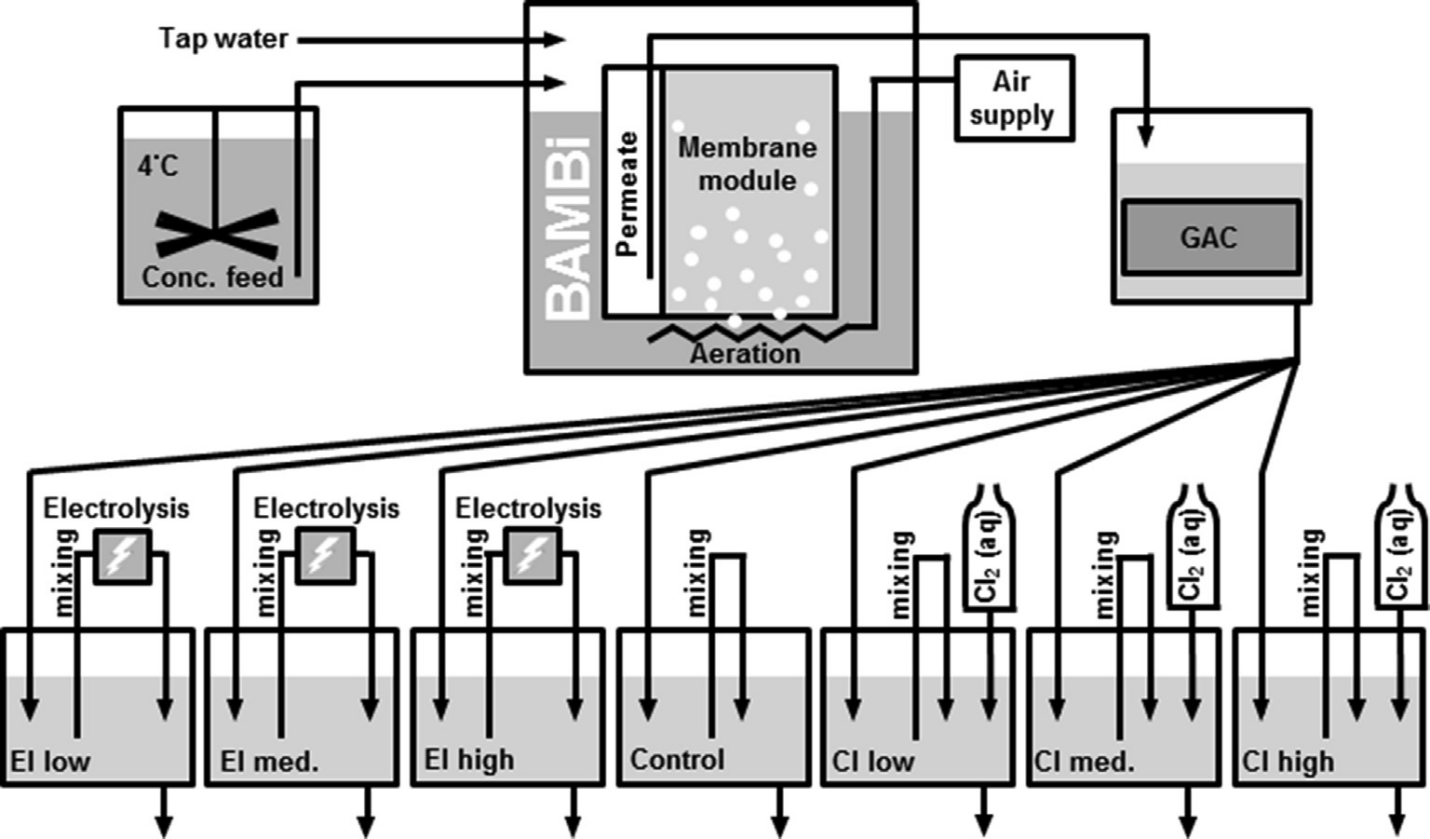
Schematic of BAMBi System. Liquid chlorine solutions [Cl_2_ (aq)] were also stored at 4 °C. Laboratory pumps were used for feeding of tap water and concentrated feed, transfer of permeate to GAC tank, transfer from GAC to seven post-treatment tanks and addition of chlorine. All seven post-treatment tanks were mixed by constant recirculation at 0.5 L/min.

A BAMBi system (Fig. 1) has been constructed using a 52 L plastic tank and standing sandwich membrane module (Microclear MCXL module, Newterra, Ontario, Canada) with 3 m^2^ total surface area polyethersulfone ultrafiltration membrane (Microdyn-Nadir, Wiesbaden, Germany). Aeration (20 L/min) was delivered with coarse bubbles (∼3 mm diameter) introduced directly below the membrane module. An interruption in the aeration was discovered on the 43rd day of operation, in which the air supply had been unintentionally disconnected. The aeration was resumed at this time, but the duration of the outage is not known. The only pressure driving water though the membrane was generated by the water head on the feeding side (approximately 35 mbar on average).

The BAMBi was fed 75 L/day of a synthetic greywater solution (Section 2.2). The daily feed was a combination of 1 part of refrigerated (4 °C) 20X concentrated solution (3.75 L/day) and 19 parts chlorine-free tap water (71.25 L/day). This total daily volume of 75 L/day was divided into 50 simulated hand washing events (1.5 L/event), spaced evenly throughout the day. The permeate from this reactor was then pumped (at 5-min intervals) to a submerged granular activated carbon (GAC) filter containing 8 L of Norit 830 coal-based GAC with 1–1.3 mm grain size (Cabot, Boston, MA, USA) for an average residence time of approximately 2.5 h. The water from the GAC tank was pumped (constantly) into 7 different post-treatment tanks, each with a volume of 10 L and a residence time of 1 day. The GAC tank and the post-treatment tanks used overflows to maintain volume. GAC was included in this system to remove organics from the water through a combination of sorption and biological degradation. The improvements in water quality produced by a BAMBi system following the addition of a GAC filter have been documented previously for a wastewater similar in composition to our nutrient-balanced hand washing water (Nguyen et al., 2017). Three post-treatment tanks were dosed with different concentrations of chlorine and three with different dosages of electrolysis. The seventh tank was a control that did not receive chlorine or electrolysis treatment. Feeding, permeate removal and chlorine addition pumps, electrolysis power and electrolysis polarity switching were activated and deactivated using process control hardware (Endress + Hauser AG Reinach BL, Switzerland), and automation softwares (Codesys, 3S-Smart Software Solutions GmbH, Kempten, Germany and CitectSCADA, Schneider Electric, Rueil-Malmaison, France). Electrolysis and chlorination controls are explained in Section 2.3.

### 2.2 Synthetic hand washing water feeding

The recipe for the synthetic feed was taken directly from, and is described in great detail by, Ziemba et al. (2018). Table 1 presents the components added to this feed at their final (1X) concentrations. The 1X strength of feed represents the loading of soap and other materials introduced into the corresponding volume of water during hand washing. The feed was prepared as a 20X concentrated version of the concentrations in Table 1, and it was diluted to the 1X version as it was used. The concentration of sodium chloride was increased in this 1X feed to provide a total chloride concentration of 100 mg/L. The reason for this increase in sodium chloride is because we expect that repeated recycling of the same water in our system will cause the salts in the water to become concentrated over time. The concentration of chloride in the water may significantly impact the production of chlorine through electrolysis. Therefore, we have elected to conduct our testing with a potentially more-representative concentration of chloride.

**Table 1:** Recipe for 1X nutrient-balanced synthetic hand washing water prepared in tap water^a^.

Chemical name	Chemical formula	Concentration (mg/L)
Sodium dodecyl sulfate (SDS)	NaC_12_H_25_SO_4_	210
Glycerol	C_3_H_8_O_3_	75
Lactic acid	C_3_H_6_O_3_	1.1
Humic acid	C_9_H_8_Na_2_O_4_	2.6
Kaolinite (as Kaolin)	Al_2_Si_2_O_5_(OH)_4_	23
Ammonium chloride	NH_4_Cl	40
Sodium nitrate	NaNO_3_	126
Disodium phosphate dihydrate	HNa_2_PO_4_·2H_2_O	45
Sodium sulfate	Na_2_SO_4_	12
Potassium chloride	KCl	5
Iron(II) chloride tetrahydrate	Cl_2_Fe·4H_2_O	4.4
Manganese(II) chloride tetrahydrate	Cl_2_Mn·4H_2_O	0.02
Cobalt(II) chloride hexahydrate	Cl_2_Co·6H_2_O	0.002
Sodium chloride	NaCl	97

a The tap water in Zürich has been measured as containing 0.7 mg/L N, 5 mg/L S, 50 mg/L Ca, 1.2 mg/L K, 7 mg/L Mg, <0.005 mg/L P, Fe, Zn and <0.0005 mg/L Mn and Cu (City of Zürich, 2016). Mo and Co are assumed to be zero.

### 2.3 Chlorination and electrolysis post-treatments

Chlorine was dosed using peristaltic pumps from refrigerated working solutions (4 °C) of 125, 400 and 1200 mg/L Cl_2_ (aq) for low (0.2 mg/L), medium (1 mg/L) and high (5 mg/L) final free available chlorine concentrations. The chlorine working solutions were prepared in chilled (4 °C) de-ionized (DI) water with final pH adjusted to 11 using NaOH. Every 15 min, about 0.6 mL of working solution was dosed to each tank. The average flow rate of chlorine solution was approximately 0.6% of the flow rate of water entering each post-treatment tank. Water in control and chlorination tanks were mixed by constant recirculation pumping at a rate of 0.5 L/min.

Boron-doped diamond (BDD) electrolysis flow cells were employed for electrolysis treatment, with 85 cm^2^ surface area parallel-plate electrodes and a 3-mm separation distance (WaterDiam, Delémont, Switzerland). Water was pumped from the post-treatment tank, passed through an electrolysis cell and then returned to the post-treatment tank at a rate of 0.5 L/min. Electrolysis was operated at 0.3 A and 6 V with continuous on/off cycles of approximately 1/89, 1/14, and 1/4 (minutes on)/(minutes off) for low, medium and high doses, respectively. The amperage was selected though trial and error such that the all target doses could be achieved with the same amperage without imposing too lengthy off times for the lowest dose. Unintended variation occurred in electrolysis dosing between days 47 and 57 of operation. The degree of electrolysis dosage that occurred during this period is unknown. Proper control was resumed on day 57 and dosage rates were intentionally modified to better achieve the target chlorine concentrations in each tank. Data collected between (not including) days 47 and 57 for electrolysis treatment and all data from days 43 and 44 (when an aeration interruption occurred in BAMBI) were not included in statistical analysis. Monitoring of chlorine concentrations, resulting from the direct chlorination and the electrolysis treatment, suggested that stable dosages of electrolysis resulted in increasing free chlorine production over time. The on/off times were modified to return the free chlorine residual concentrations to the desired targets. The dosing was reduced 6.7% for the medium dose and 33% for the high dose on day 57. The medium dose was reduced an additional 10% and the low dosage was increased 19% on day 61. The polarity of the current was reversed between each on/off cycle. Recirculation pumps were operated continuously. Electrolysis cells were examined for fouling every 1–2 weeks during testing, but no significant fouling was observed.

### 2.4 Chemical analysis

DOC was monitored using a TOC/DOC analyzer (Shimadzu TOC-L, Kyoto, Japan). Ammonium was measured with gas-diffusion flow injection (Foss, Hillerød, Demark) or Hach Lange kits (LCK 304, Hach, Loveland, Colorado, USA). Nitrite, nitrate and chloride were measured by ion chromatography (Metrohm 881, Herisau, Switzerland). Free and combined chlorine were measured using Hach Lange kits based on the DPD (*N*,*N*-diethyl-*p*-phenylenediamine) method (LCK 310, Hach).

### 2.5 Total cell counts (TCC) intact cell counts (ICC) and AOC

TCC in the post-treatment tanks and TCC for AOC measurements were conducted using SYBR^®^ Green I stain (ThermoFisher Scientific, Waltham, Massachusetts, USA) and a flow cytometer (Cytoflex, Bechman Coulter, Brea, California, USA) with 30 s reads on 30 μL samples. Intact cell counts (ICC) were conducted as with TCC, except propidium iodide (PI) was also added during incubation. Flow cytometry analysis was selected for bacterial quantification because of the simplicity and speed of processing high quantities of samples (relative to sequencing or qPCR techniques), the ability to quantify bacteria that are non-culturable (and not captured by heterotrophic plate count), and the ability to differentially identify cells with intact cell membranes (TCC) from cells with damaged cell membranes (ICC) (Hammes et al., 2011; Joux and Lebaron, 2000). Flow cytometry has also been demonstrated to descriptively quantify changes in cell counts through drinking water treatment processes (Hammes et al., 2008). The AOC measurement procedure was based on the procedure of Hammes and Egli (2005) which consisted of filtering 50 mL of sample through a 0.2 μm polyethersulfone filter (Pall Port Washington, New York, USA) into a 60 mL glass vial, adding 1 mL of a standard and diverse inoculum of environmental bacteria, dividing into three 45 mL glass vials, incubating for 3 days at 30 °C without light, measuring TCC, and then calculating AOC using the equation 1 μg AOC = 10^7^ cells. All glassware was muffled at 450 °C for 12 h, PTFE-lined vial caps were used, and filters were prewashed with 50 mL of DI water. Inoculum was prepared by combining one part each: 1) Evian water (Évian-les-Bains, France), 2) water treated by a BAMBi system fed the synthetic greywater described in this study, 3) water treated by a BAMBi system fed a mixture of soap, urine and feces, and 4) water collected from the Chriesbach stream in Dübendorf, Switzerland. Bacteria from the BAMBi waters and the stream water were twice washed and re-suspended in mineral buffer (LeChevallier et al., 1993) by centrifugations for 10 min at 3500g. After each centrifugation, the supernatant was discarded and the cells were re-suspended in the buffer. The TCC of the finished inoculum was determined to be ∼100 cells/mL. The composition of the inoculum was modified from that of Hammes and Egli (2005) because AOC measurements rely on inoculating with bacteria that are best able to grow on the carbon within the sample. Natural inoculum are often used to measure AOC in natural waters because of the diversity and because the bacteria can be well-adapted to the carbon and the background materials in the water. In engineered systems, including bacteria adapted to these environments may also improve the quality of AOC measurements.

## 3 Results

### 3.1 Direct chlorination and electrolysis generally achieved free chlorine targets

**Fig. 2:**
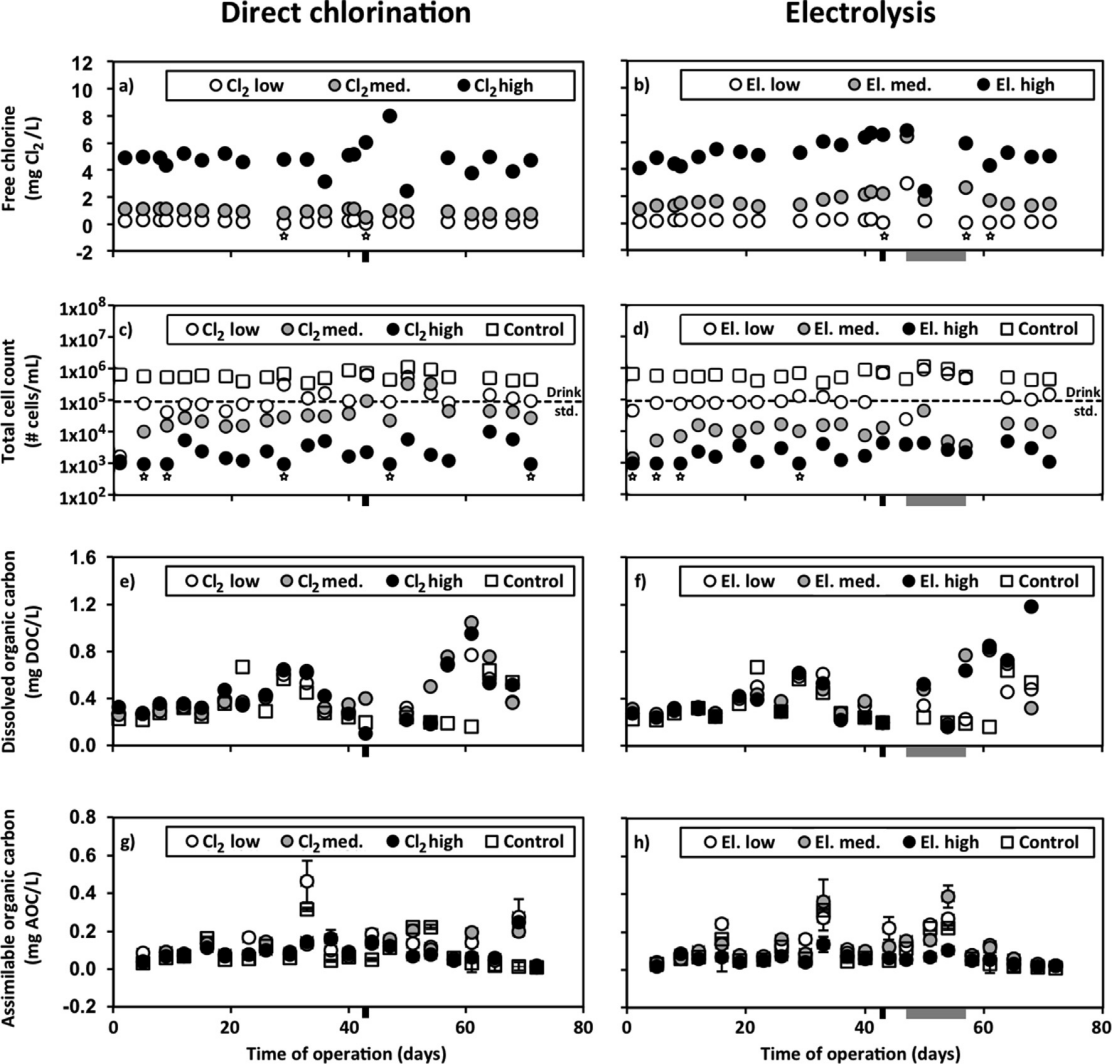
Free chlorine, total cell count (TCC), dissolved organic carbon (DOC) and assimilable organic carbon (AOC) for different dosages of direct chlorination (left plots), dosages of electrolysis (right plots) and control. The axis titles and scales are the same for horizontal pairs. All plots have the same x-axis title and scale. Measurements below quantification are plotted as the quantification limit and indicated using a star placed below the data point. Dashed lines indicate TCC of drinking water. Black bars beneath the x-axes indicate an interruption in aeration. Grey bars beneath the x-axes represent an interruption in electrolysis control.

The target residual free chlorine concentrations in the low, medium and high dose conditions were 0.2, 1 and 5 mg Cl_2_/L for chlorine dosed directly, or produced through electrolysis. In each case, the free chlorine concentrations observed were generally close to the target concentrations. For direct chlorination, the median free chlorine concentrations were 0.21, 0.95 and 4.8 mg Cl_2_/L, respectively for increasing dosages (Fig. 2a). For electrolysis, the median free chlorine concentrations were 0.21, 1.5 and 5.2 mg Cl_2_/L, respectively for increasing dosages (Fig. 2b). With the electrolysis treatment, there was a clear increase in the free chlorine concentrations over time for the first 47 days of operation in the tanks treated with medium and high dosages. The free chlorine concentration in the post-treatment tank with the highest electrolysis dosage increased over the first 47 days–0.4 mg Cl_2_/L free chlorine per week. The electrolysis modules were not controlled properly between the 47 and 57 operating days, resulting in unintended variation in the electrolysis dosing during this period, resulting in free chlorine concentrations ranging from below detection to 3 mg Cl_2_/L in the low dose, from 1.7 to 6.4 mg Cl_2_/L in the medium dose, and 2.4–6.9 mg Cl_2_/L in the high dose. Starting on day 57, proper control was resumed, and the on/off times were modified to achieve the initial (target) chlorine concentrations for each dosage.

### 3.2 Increasing chlorine residual reduced TCC and ICC

**Fig. 3:**
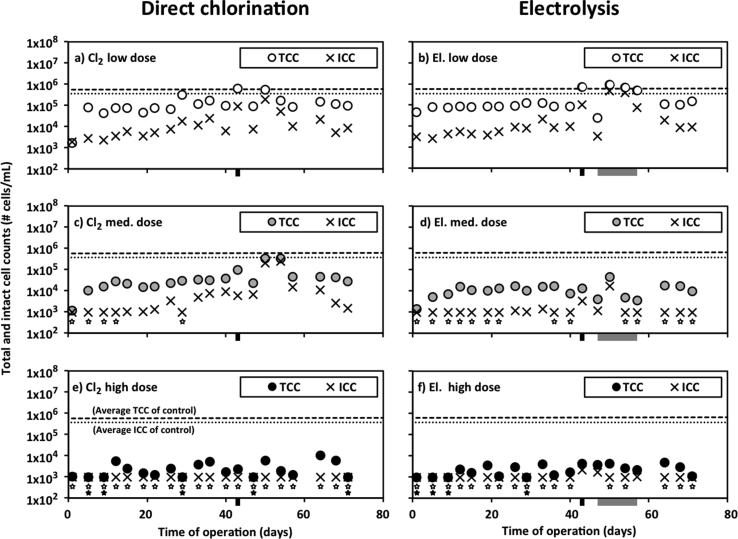
Total cell count (TCC) and intact cell count (ICC) resulting from three different dosages of direct chlorination or electrolysis treatments. Dashed lines indicate the average TCC of the control tank. Dotted lines indicate the average ICC of the control tank. Measurements below quantification are plotted as the quantification limit and indicated using a star placed below the data point. White-filled starts refer to TCC data and black-filled stars refer to ICC data. Black bars beneath the x-axes indicate an interruption in aeration. Grey bars beneath the x-axes indicate an interruption in electrolysis control.

The TCC includes all cells that can be identified by the flow cytometer. TCC and ICC results are presented in Fig. 2c and d, individually in Fig. 3 and in Supporting Information Tables S1 and S2. The median TCC for the control tank was 5.6 × 10^5^ cells/mL. For increasing concentrations of direct chlorination, the median TCC values were 9.0 × 10^4^, 2.8 × 10^4^ and 1.6 × 10^3^ cells/mL, respectively. For increasing dosages of electrolysis treatment, the median TCC values were 8.6 × 10^4^, 1.0 × 10^4^ and 1.7 × 10^3^ cells/mL, respectively. Direct chlorination and electrolysis treatments produced similar TCC values for low, medium and high residual free chlorine concentrations, respectively.

The TCC of the water from the GAC filter that was continuously added to each post-treatment tank, was characterized over a 4 day period to have a median and average of 3.1 × 10^5^ cells/mL with a standard deviation of 5 × 10^4^ cells/mL (data in Supporting Information Table S3), which is similar to, but lower than, the concentration observed in the control tank. The TCC of all tanks dosed with chlorine or electrolysis were generally less than the TCC of the water entering each tank. The TCC observed in drinking water in the city of Zürich, Switzerland is also included in Fig. 2c and d, at 8.97 × 10^4^ cells/mL (Hammes et al., 2010). The TCC of the control tank that does not include any post-treatment exceeded this drinking water concentration on every day sampled. The median TCC values for the low dosages of direct chlorination and electrolysis match this drinking water value. The daily TCC values for the high dosage of direct chlorination and both the medium and high dosages of electrolysis were consistently below the drinking water concentration and the medium direct chlorination TCC was below the drinking water concentration in 18 out of 20 samples.

ICC in this study refers to a count of cells which are successfully stained by a total cell stain, but cannot be stained by PI, because PI is only able to enter cells with damaged cell membranes. ICC results for each tank are presented in Fig. 3 and in Supporting Information Table S2. In general, our results indicate that lower doses of chlorination and electrolysis treatments caused greater log reductions in ICC relative to TCC, and high doses produced similar reductions in TCC and ICC. The median value for the ICC/TCC ratio was 67% (average is 70%) for the control tank. This was similar to the ∼58% ICC/TCC ratio observed in the water entering the post-treatment tanks from the GAC filter. The median ICC/TCC ratios for low doses were 8.5% (16% average) for direct chlorination and 6.7% (8.8% average) for electrolysis. ICC data becomes limited for medium and high doses of chlorine and electrolysis because the ICC concentrations were frequently (or almost exclusively) below the quantification limit. For the samples in which the ICC/TCC ratio can be enumerated using values above the quantification limit, the ratio may increase with increasing dosage, to 19% and ICC/TCC median values for medium chlorination (high chlorination produced no quantifiable ICC values), and 9.2 and 46% ICC/TCC median values for medium and high electrolysis treatments.

### 3.3 DOC and AOC may both be affected by chlorine and electrolysis treatments

The DOC concentrations measured in control and post-treatment tanks are presented in Fig. 2e and f. The DOC of all tanks increased or decreased together at different sampling times, possibly due to variation in the water produced by the BAMBi + GAC filter system. The median DOC of the control tank was 0.28 mg DOC/L, with a range from 0.16 to 0.67 mg DOC/L. The median DOC values for increasing direct chlorination were slightly higher at 0.34, 0.37 and 0.39 mg DOC/L, respectively, and for increasing electrolysis, 0.34, 0.38 and 0.35 mg DOC/L, respectively. Rank analysis of individual sampling days indicated that the DOC concentration of the control tank was lower than of the direct chlorination tanks for 12 out of 18 samples (13 out of 18 including equal values) and lower than electrolysis tanks 8 out of 16 times (11 out of 16 including equal values). Friedman testing between either the control and the three dosages of direct chlorination, or between the control and three dosages of electrolysis indicated that each ranking was not the result of random variation at a greater than 99.5% confidence level. Therefore, the lower DOC in the control tank was statistically significant compared to tanks that received chlorine or electrolysis. It was difficult to discern clear relationships for DOC rank between three dosages of direct chlorination or between the three dosages of electrolysis. In each case, low, medium and high doses produced both highest and lowest DOC values during the experiment, resulting in similar average ranks ranging between 1.75 and 2.16.

The concentrations of AOC in control and post-treatment tanks are presented in Fig. 2g and h. The AOC concentrations in different post-treatment tanks would generally increase or decrease together at different sampling points, suggesting an influence of variation in the influent water. A similar increasing or decreasing together was apparent in DOC concentrations, but AOC concentrations were not necessarily correlated with DOC concentrations. The median AOC value of the control was 0.059 mg AOC/L with a range from 0.006 to 0.315 mg AOC/L. The median AOC values for increasing direct chlorination dosages were 0.096, 0.091 and 0.077 mg AOC/L, respectively. The median AOC values for increasing electrolysis dosages were 0.080, 0.086 and 0.054 mg AOC/L. The values for direct chlorination and for electrolysis were generally comparable, with each producing values for low and medium dosages that are higher than the control, and then high dosages that are more similar to the control. Rank analysis of individual sampling days indicated that the AOC concentration of the control was lower than the AOC of the three direct chlorination tanks 13 out of 29 times, and lower than the three electrolysis tanks 7 out of 17 times. The highest dosage of direct chlorination produced the lowest AOC concentration out of all three electrolysis tanks and the control, 7 out of 17 times, and electrolysis produced the lowest AOC the remaining 10 times. Friedman testing between either the control and the three dosages of direct chlorination, or between the control and three dosages of electrolysis indicated that the ranking of each set is not the result of random variation at a greater than 97.5% confidence level.

### 3.4 Disruptions in the system impact anti-bacterial performance

**Fig. 4:**
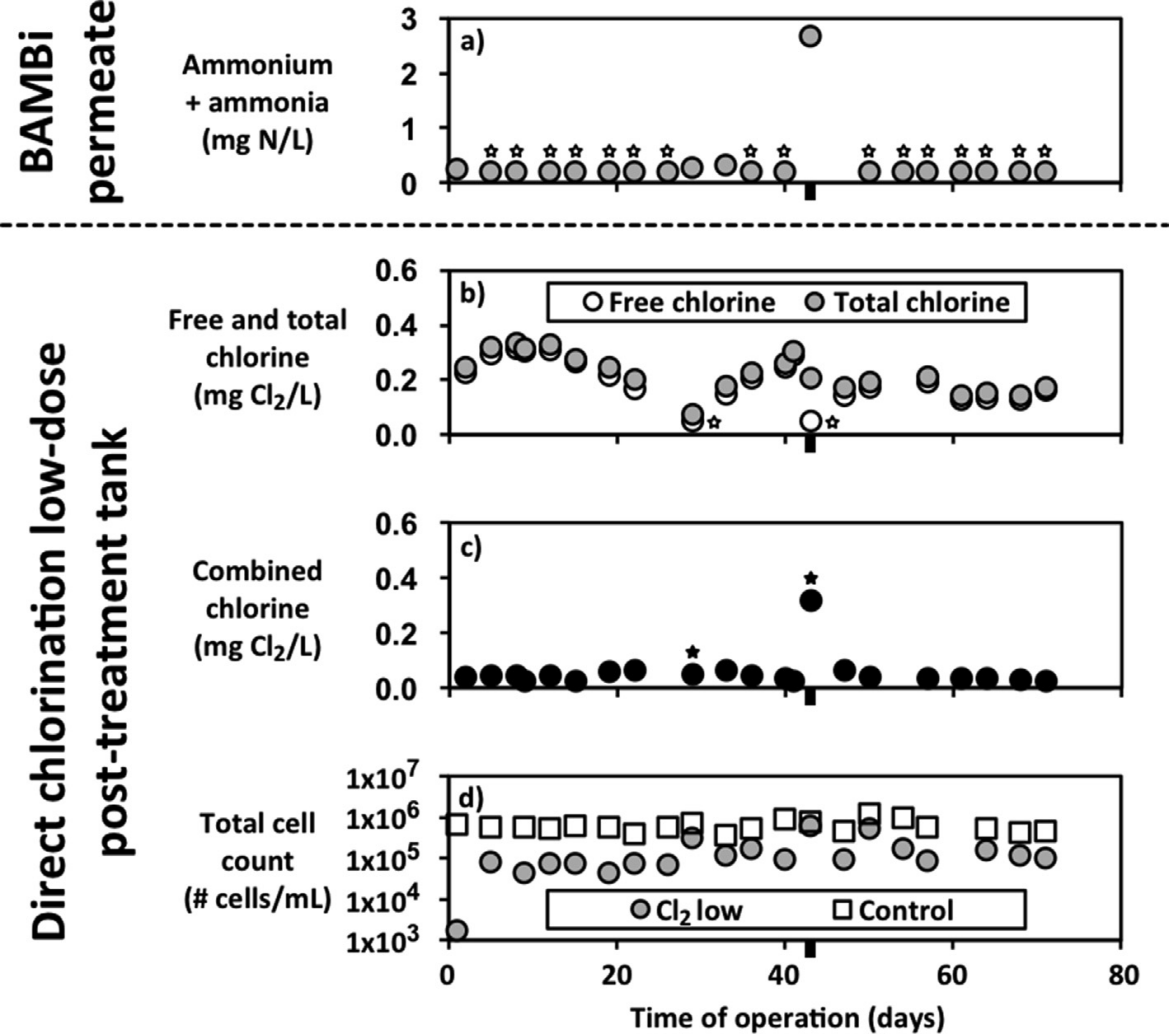
BAMBi permeate quality (a) is linked to chlorine (b, c) and TCC (d) concentrations in the low dose direct chlorination tank. Measurements below quantification are plotted as the quantification limit and indicated using a white-filled star placed below the data point. Black-filled stars are placed below values calculated using the quantification limit as the free chlorine value. Black bars beneath the x-axis indicate an interruption in aeration.

Monitoring both the chemical composition of BAMBi permeate and the chemical and microbial composition of water in each post-treatment tank allows us to observe how variation and disruption in BAMBi can impact the safety of the finished water product. Fig. 4 illustrates this connection based on a disruption that occurred on day 43 of testing. Fig. 4a presents the combined concentrations of ammonium and ammonia (ammonium + ammonia) in the permeate water produced by the BAMBi. On day 43, a disruption occurred, likely linked to a temporary interruption in aeration, resulting in a failure of nitrification within the BAMBi and a spike in the concentration of ammonium + ammonia in the permeate to 2.7 mg N/L. Fig. 4b demonstrates that there was variation in the concentrations of free and total chlorine in the low dose, direct chlorination tank, but shows how the free and total chlorine concentrations were quite similar under normal operating conditions. Total chlorine is the combination of unreacted free chlorine, and free chlorine that has reacted with ammonia or ammonia-containing organics. On day 43, there is a clear drop in free chlorine to less than 0.05 mg Cl_2_/L (below quantification limit) while the total chlorine meets the expected 0.2 mg Cl_2_/L target value. Fig. 4c plots the concentration of combined chlorine, displaying the spike on day 43 due to the spike in ammonium + ammonia in the system. Fig. 4d shows that the reduced concentration of free chlorine has allowed bacteria to increase to a TCC value of 6.1 × 10^5^ cells/mL, which is significantly higher than the median value over the course of the experiment (9.0 × 10^4^ cells/mL), and higher than the concentration of Zürich drinking water (8.97 × 10^4^ cells/mL) (Hammes et al., 2010).

## 4 Discussion

The discussion is divided into three sections. The first section focuses on understanding the anti-bactreial mechanisms that produce the reductions in bacterial concentrations observed with both direct chlorination and electrolysis treatments. The second section discusses why we do not see significant differences between direct chlorination and electrolysis treatments operating at similar concentrations of chlorine residual. The third section looks specifically at how the different dosages of direct chlorination and electrolysis treatment perform with respect to bacterial concentrations and how this work advances our ability to design water recycling systems that ensure microbial safety.

### 4.1 Direct inactivation by chlorine is the dominant anti-bacterial mechanism

The three potential anti-bacterial mechanisms that we might expect to observe with direct chlorination or electrolysis treatments are: (1) direct inactivation of bacteria, (2) direct growth inhibition of viable bacteria, or (3) indirect growth inhibition by removal of carbon substrate. Our results demonstrate that inactivation is the dominant anti-bacterial mechanism. In every experimental condition tested, we observed a net reduction in TCC and ICC of at least one order of magnitude compared to the bacterial concentrations of the water entering each tank. We cannot quantify from our data to what degree some growth might still occur in the presence of the different chlorine residual concentrations, however any growth that does occur is less significant than the amount of inactivation. The potential for growth in the presence of chlorine is likely significantly less than the potential for growth in the control, which is itself quite small (population doubles over 24-hour residence time). A specific comparison between direct chlorination and electrolysis treatments is provided in Section 4.2. Results display similar concentrations of TCC, ICC, DOC and AOC as a function of free chlorine residual. These results do not indicate that TCC, ICC, DOC or AOC concentrations are differentially affected by direct chlorination or electrolysis treatments if the concentration of free chlorine residual is the same. This similarity in performance leads us to attribute all significant inactivation of bacteria and chemical reactions with organic compounds to chlorine, and not to any other oxidants that may or may not be produced by the electrolysis treatment. Therefore, we find that the dominant anti-bacterial mechanism in our system is inactivation and we credit this inactivation solely to the presence of chlorine.

We have now described reductions in terms of both TCC and ICC as inactivation, though we would calculate dramatically different values of inactivation by each measure. What constitutes life or death (or inactivation) in bacteria is a complex scientific question, as is how best to measure the concentration and viability of cells (Buysschaert et al., 2016; Prest et al., 2016). The FCM protocols for TCC and ICC (utilized in this study) rely on staining nucleic acids within each cell (Hammes et al., 2011). TCC stains such as SYBR^®^ Green I are generally able to permeate through a cellular membrane, access and then stain the nucleic materials within a cell. PI is used for ICC staining because it is less permeable through the cellular membrane. Positive staining with PI, indicates cells that have suffered membrane damage. Cells which stain positive with a TCC stain but do not stain positive with a membrane integrity stain (like PI) are generally considered intact. Reduction in ICC is often credited as being a conservative indicator of losing viability because a cell must usually undergo significant membrane damage before it becomes permeable to the PI stain (Hammes et al., 2011; Joux and Lebaron, 2000). More extensive damage to a cell can result in the destruction of the nucleic materials within the cell, which prevents positive enumeration as TCC. Sufficient destruction of nucleic materials to produce a reduction in TCC concentration has been observed previously, following exposure to ozone (Ramseier et al., 2011b). In our study, the reductions in TCC observed at low, medium and high concentrations of chorine residual demonstrate a similar oxidation of the cells by chlorine. This degree of oxidation is beyond the amount required to cause permanent inactivation, as indicated by ICC measurements. The changes in ICC concentration likely provide a more accurate measure of permanent bacterial inactivation in our system, compared to the changes in TCC.

The work of Mao et al. (2018) presents a similar investigation of long-term changes in TCC and ICC due to chlorination. Over five days of exposure to 0.3 mg Cl_2_/L, *Pseudomonas aeruginosa*, in simulated drinking water, displayed reductions of 4 or more log in ICC and approximately 1 log in TCC. These results are comparable with the moderate reductions in TCC and the much more significant reductions in ICC observed in our study. *Pseudomonas aeruginosa* inactivation was conducted in pure culture, while our study utilized a community of bacteria. The presence of a community of bacteria introduces the possibility that some bacteria in our system may be inactivated in chlorine at faster, comparable, or slower rates than the *Pseudomonas aeruginosa* tested by Mao et al. (2018). TCC and ICC measurements cannot easy discrimination between subpopulations of bacteria. This means that TCC and ICC measurements cannot identify if some sub-populations of bacteria are able to survive or grow as the total population decreases. The results do demonstrate that in tanks fed the same diverse population of bacteria, increasing chlorine or electrolysis exposure is able to reduce the populations of all bacteria to below detection.

Our results do not indicate a clear difference in DOC concentrations between tanks with low, medium and high doses of direct chlorination or electrolysis treatments, but all experimental tanks have higher DOC than the control. This behavior is consistent with a control tank where some DOC is used for growth and the DOC is not impacted in the other tanks where we do not expect significant growth. The AOC results suggest similarly, that AOC was consumed by growth in the control, but that AOC was also consumed by reacting with chlorine at the highest residual concentration tanks (∼5 mg Cl_2_/L). Previous research has indicated that chlorine oxidation is capable of transforming a portion of the DOC to AOC in drinking water (Liu et al., 2015), but alternative evidence suggests that chlorination can also reduce AOC concentrations (Ramseier et al., 2011a). An increase or a decrease in AOC with chlorination is likely driven by the specific forms of AOC and DOC interacting with the chlorine. In our study, a high free chlorine residual results in an AOC reduction, and this reduction may also impact the ability of bacteria to grow in the water. Biological stability is a concept for microbial control, through which the concentrations of AOC or other nutrients are sufficiently low that bacterial growth within the water is prevented or extremely limited (Prest et al., 2016; van der Kooij, 2000). The threshold for biological stability has been suggested to occur at approximately 0.01 mg AOC/L (Prest et al., 2016; van der Kooij, 2000). The median AOC values we observed in our system ranged from 5 to 11 times this threshold. Independent of nutrient availability and how chlorine might affect growth, the dominant anti-bacterial mechanism observed in our system remains inactivation by chlorine.

### 4.2 Anti-bacterial performance of electrolysis matches the performance of direct chlorination for a given free chlorine concentration

Electrolysis treatment is capable of producing hydroxyl radicals (Martínez-Huitle et al., 2015), and hydroxyl radicals are capable of both mineralizing organic materials to CO_2_ (Martínez-Huitle et al., 2015; von Gunten, 2003) and directly inactivating bacteria (Jeong et al., 2006). The electrolysis treatment in this current study, did not clearly impact TCC, ICC, DOC or AOC concentrations beyond the influence attributed to the free chlorine the electrolysis did produce. Studies utilizing BDD electrodes on drinking water have also found that chlorine production is the dominant disinfection mechanism (Kerwick et al., 2005; Kraft et al., 1999).

Many parameters control the chemical effects of electrolysis treatment, including the potential, current density, material and orientation of the electrodes; the characteristics of the water being treated; and the mass transport conditions within the electrolysis cell (Martínez-Huitle et al., 2015). In this study it is likely that the water quality significantly impacted the dominance of chlorine production over carbon mineralization. Unlike concentrated wastewater sources, the DOC in our water, following treatment with BAMBi and GAC, contains only ∼0.4 mg DOC/L. The chloride concentration in the water was set at 100 mg Cl^−^/L. This chloride concentration is 1–2 orders of magnitude higher than the content of drinking water, however this increase was designed to reflect water that had been recycled in the BAMBi system over and over again, as we assume that repeated uses of the water will lead to salt accumulation. The high ratio of chloride to DOC compounds strongly favors chlorine production simply because there are more interaction opportunities for oxidation with the chloride. Experimental testing and electrochemical theory suggest that lower flow rates and higher current densities can promote mineralization over chlorine production (Scialdone et al., 2009). While it is difficult to productively compare the flow rate used in this study with the flow rates tested by Scialdone et al. (2009), because the configuration of the flow cell must also be considered, both current density vales (17, 39 mA/cm^2^) examined by Scialdone et al. (2009) were significantly higher than what we tested in this study (3.5 mA/cm^2^). Increasing the current density in our application might better promote mineralization, but this might not overcome the influence of the chloride/DOC ratio of our water. Another study seeking to optimize BDD electrode disinfection, this time in treated wastewater, found that increasing current density incurred greater energy costs but did not improve inactivation of bacteria, (Schmalz et al., 2009). Once again, chlorine oxidation was identified as the dominant mechanism of inactivation (Schmalz et al., 2009).

### 4.3 Implementing chlorination or electrolysis to achieve microbial safety

There is not a scientific consensus for what must be done to water before it is safe for hand washing. While it might not be necessary for hand washing water to reach the same level of microbial safety as drinking water, drinking water quality could be considered as a conservative goal for recycled hand washing water. Drinking water is frequently used for hand washing in many well-developed countries and is generally regarded as safe for human exposure. In terms of organic carbon, our BAMBi + GAC treatment system achieved >99% removal of DOC and ultimately achieved a DOC concentration consistent with drinking water levels (0.05 mg DOC/L) (City of Zürich, 2016). Chlorination proved to be an effective strategy to reduce bacteria concentrations and to combat bacterial growth in our system as it has in municipal drinking water distribution networks. When our system achieved stable operating conditions, even the lowest doses (0.2 mg Cl_2_/L residual), of both direct chlorination and electrolysis treatment, generally matched the TCC concentration (8.97 × 10^4^ cells/mL) in drinking water in Zürich Switzerland (Hammes et al., 2010). A detailed investigation of TCC concentrations throughout a municipal drinking water treatment plant concluded that changes in TCC concentrations described well the impacts of the main treatment processes (Hammes et al., 2008). We expect that monitoring TCC also effectively captures the impacts of treatments in our smaller-scale system. The ICC concentration in our system, which better reflects viable bacteria, was less than 10% of the TCC concentration. There is some risk that reductions in TCC and ICC do not reflect a chlorine-resistant population that may persist during treatment. This possibility could be explored in future testing, yet the extreme destruction of cells (resulting in reductions in TCC) that we observed, even at our lowest residual chlorine concentrations, suggests an extremely difficult environment for potential survivors. While we did observe effective inactivation at 0.2 mg Cl_2_/L residual, operating under stable conditions, our testing indicated that chlorine residual targets should be designed to overcome more challenging operating conditions. This concept is particularly important for small-scale decentralized water recycling systems and particularly important for developing world applications. The unintentional disruption in BAMBi aeration we experienced during our testing resulted in the tank with the lowest free chlorine residual (0.2 mg Cl_2_/L) failing to maintain TCC values below the drinking water concentration, while the medium and high chlorine dosing still maintained enough chlorine residual to keep the TCC below drinking water concentration.

The results from variation and the unintended disruption observed in this study indicate that a free chlorine residual target of 1 mg Cl_2_/L would be an effective treatment in our system. This residual chlorine target of 1 mg Cl_2_/L is a common design target or minimum threshold for many laboratory and case studies, and has generally been observed to be sufficient anti-bacterial control by different means of testing (Abdel-Kader, 2013; Friedler et al., 2005; Gual et al., 2008; March et al., 2004; Winward et al., 2008). These studies have not attempted to deliver drinking water or hand washing water quality.

The chief limitations of this study are based on not understanding the specific types and risks of bacteria in the system, how these bacteria are affected by chlorination under dynamic conditions, and how this relationship might change under realistic and challenging operating conditions. A better-informed strategy for target chlorine residual concentration should ultimately be based on a risk assessment that incorporates each of these considerations. Reliance on TCC concentrations alone introduces the possibility that lower concentration of a more-harmful bacteria might present a greater risk than a higher concentration of a less-harmful bacteria. Sequencing or qPCR techniques could be used to identify all bacteria or specific targets. This study details the interactions between water constituents, free chlorine and bacteria concentrations that were observed during an unintended disruptive event. Systematic testing examining realistic and intentional disruptive events with dynamic monitoring of relevant chemical species and bacteria concentrations would demonstrate if higher chlorine concentrations are needed, but also what disruptive events may simply overload the system. Optimization of operational parameters (i.e. aeration, residence time, recirculation rate or nutrient-balance) may mitigate demands on chlorine, and these factors must also be considered. Though this study examined a residual chlorine concentration of 5 mg Cl_2_/L, the upper boundary for a real system might be 4 mg Cl_2_/L, which is the maximum residual disinfectant level for chlorine in drinking water (US EPA, 2009). Beyond this concentration the EPA notes potential eye and/or nose irritation, which is a consideration that could also apply for hand washing applications (US EPA, 2009).

Our results demonstrate that sufficient quantities of chlorine can be delivered by either direct chlorination or produced through electrolysis. We have discussed that it may be possible to optimize electrolysis to promote mineralization of carbon and possible removal of AOC, however it is not clear if this potential anti-bacterial mechanism provides a benefit in a system that is currently dominated by destructive oxidation of the bacteria. A more productive strategy could be to better understand the relationship between electrolysis and chlorine production in our system. We have manually adjusted the dosages of electrolysis treatment to achieve our target residual concentrations to compensate for an apparent increase over time in chlorine production efficiency. A thorough understanding of what caused this increase would make the application of electrolysis more accurate and require less monitoring. Automatic feedback on electrolysis or direct chlorination could allow the dosing of either post-treatment to better adapt to variations or disruptions and perhaps allow the chorine residual target to be < 1 mg Cl_2_/L. This study has demonstrated that both chlorine and electrolysis treatments complement well our primary BAMBi + GAC treatment to deliver hand washing water that achieves drinking water quality in terms of DOC and TCC concentrations. Choosing between direct chlorination or electrolysis treatments, and possible inclusion of any feedback dosing, should likely be done in accordance with cost and service constrains within specific applications.

## 5 Conclusions

• The combined treatment of a BAMBi, GAC and either direct chlorination or electrolysis disinfection is suitable to reduce DOC and TCC concentrations from hand washing water to values below drinking water concentrations in Zürich, Switzerland.
• The electrolysis treatment resulted in the production of chlorine and concentrations of TCC, ICC, DOC and AOC similar to systems in which the same concentration of chlorine was added directly. This indicates that bacterial inactivation through oxidation by chlorine was the dominant anti-bacterial mechanism and that more powerful oxidants such as hydroxyl radicals were either not produced or did not have any impact on the concentration or bioavailability of carbon or bacterial inactivation.
• The dosing of chlorine through direct chlorination or electrolysis must include a factor of safety such that a free chlorine residual is maintained during challenging and disruptive operation.

## Author declaration

All authors confirm that the manuscript has been read and approved by all named authors and that there are no other persons who satisfied the criteria for authorship but are not listed. We further confirm that the order of authors listed in the manuscript has been approved by all of us. We confirm that we have given due consideration to the protection of intellectual property associated with this work and that there are no impediments to publication, including the timing of publication, with respect to intellectual property. In so doing we confirm that we have followed the regulations of our institutions concerning intellectual property. We understand that the Corresponding Author is the sole contact for the Editorial process (including Editorial Manager and direct communications with the office). He is responsible for communicating with the other authors about progress, submissions of revisions and final approval of proofs. We confirm that we have provided a current, correct email address which is accessible by the Corresponding Author and which has been configured to accept email from.

## Declaration of interests

The authors declare that they have no known competing financial interests or personal relationships that could have appeared to influence the work reported in this paper.
