# Supplementary material for: Comparing the anti-bacterial performance of chlorination and electrolysis post-treatments in a hand washing water recycling system
Source: Water Res X. 2018 Dec 14;2:100020. doi: 10.1016/j.wroa.2018.100020 (PMC6510329; doi:10.1016/j.wroa.2018.100020)
Supplement: Supporting Information [file mmc1.docx]

**Comparing the anti-bacterial performance of chlorination and electrolysis**

**post-treatments in a hand washing water recycling system**

**Supporting Information**

Christopher Ziemba ^a, b,^ *, Odile Larivé ^a, c^, Svenja Deck ^a^,

Theo Huisman ^a, b^, and Eberhard Morgenroth ^a, b^

^a^ Eawag: Swiss Federal Institute of Aquatic Science and Technology, 8600 Dübendorf, Switzerland

^b^ ETH Zürich, Institute of Environmental Engineering, 8093 Zürich, Switzerland

^c^ (current address) EPFL Lausanne, Environmental Chemistry Laboratory, 1015 Lausanne, Switzerland

* Corresponding author. E-mail address: Christopher.Ziemba@eawag.ch

Submitted to Water Research on 26-September-2018

Revision submitted 24-November-2018

Table S.1. Total cell counts (TCC) for low, medium and high doses of chlorine; low, medium and high doses of electrolysis; and one control tank that receives no post-treatment. < quant. indicates values which are below the detection level of 970 #/mL, st. dev. indicates standard deviation.

|  | Total cell counts for each post-treatment condition (#/mL) | | | | | | | |
| --- | --- | --- | --- | --- | --- | --- | --- | --- |
| day | Cl2 Low | Cl2 Med. | Cl2 high | Control | El. Low | El. Med. | El. High |  |
| 1 | 1'670 | 1'120 | 1'020 | 673'100 | 46'890 | 1'420 | < quant. |  |
| 5 | 81'290 | 10'410 | < quant. | 578'500 | 82'280 | 5'020 | < quant. |  |
| 9 | 42'390 | 15'720 | < quant. | 553'600 | 76'410 | 7'190 | < quant. |  |
| 12 | 74'790 | 27'270 | 5'340 | 548'100 | 85'230 | 16'220 | 2'320 |  |
| 15 | 75'980 | 21'460 | 2'470 | 607'400 | 81'440 | 10'890 | 1'590 |  |
| 19 | 45'520 | 14'770 | 1'510 | 573'900 | 83'030 | 10'010 | 3'470 |  |
| 22 | 72'880 | 16'090 | 1'210 | 405'200 | 85'790 | 12'890 | 1'090 |  |
| 26 | 67'110 | 23'570 | 2'420 | 564'400 | 89'960 | 17'040 | 2'960 |  |
| 29 | 321'490 | 29'290 | < quant. | 712'600 | 127'530 | 10'460 | < quant. |  |
| 33 | 117'870 | 33'870 | 3'870 | 362'400 | 125'680 | 15'410 | 3'920 |  |
| 36 | 169'590 | 31'690 | 5'270 | 514'200 | 85'860 | 16'310 | 1'260 |  |
| 40 | 96'810 | 38'810 | 1'640 | 878'720 | 86'560 | 7'470 | 1'710 |  |
| 43 | 607'490 | 93'460 | 2'270 | 771'700 | 713'740 | 13'270 | 4'310 |  |
| 47 | 90'290 | 23'540 | < quant. | 445'400 | 23'770 | 3'990 | 3'710 |  |
| 50 | 548'520 | 343'220 | 5'690 | 1'193'700 | 918'790 | 45'610 | 4'270 |  |
| 54 | 172'100 | 324'500 | 1'920 | 949'000 | 654'100 | 4'760 | 2'520 |  |
| 57 | 87'340 | 46'230 | 1'260 | 554'000 | 500'290 | 3'610 | 2'190 |  |
| 64 | 153'380 | 45'360 | 10'220 | 522'050 | 112'510 | 17'360 | 4'840 |  |
| 68 | 115'440 | 42'310 | 5'760 | 418'900 | 102'190 | 16'530 | 2'940 |  |
| 71 | 98'170 | 28'420 | < quant. | 457'800 | 145'910 | 9'560 | 1'070 |  |
| average | 152'006 | 60'556 |  | 614'234 | 211'398 | 12'251 |  |  |
| st. dev. | 160'055 | 95'408 |  | 203'858 | 259'644 | 9'342 |  |  |
| median | 93'550 | 28'855 | 2'420 | 559'200 | 88'260 | 10'675 | 2'730 |  |

Table S.2. Intact cell counts (ICC) for low, medium and high doses of chlorine; low, medium and high doses of electrolysis; and one control tank that receives no post-treatment. < quant. indicates values which are below the detection level of 970 #/mL, st. dev. indicates standard deviation.

|  | Intact cell counts for each post-treatment condition (#/mL) | | | | | | | |
| --- | --- | --- | --- | --- | --- | --- | --- | --- |
| day | Cl2 Low | Cl2 Med. | Cl2 high | Control | El. Low | El. Med. | El. High |  |
| 1 | 1'940 | < quant. | < quant. | 425'265 | 3'140 | < quant. | < quant. |  |
| 5 | 2'820 | < quant. | < quant. | 441'715 | 2'590 | < quant. | < quant. |  |
| 8 | 2'340 | 790 | < quant. | 423'410 | 4'210 | < quant. | < quant. |  |
| 12 | 3'520 | < quant. | < quant. | 352'710 | 5'340 | < quant. | < quant. |  |
| 15 | 5'910 | 1'040 | < quant. | 426'340 | 4'090 | < quant. | < quant. |  |
| 19 | 3'510 | 1'010 | < quant. | 572'385 | 3'710 | < quant. | < quant. |  |
| 22 | 5'210 | 1'320 | < quant. | 384'005 | 5'320 | < quant. | < quant. |  |
| 26 | 7'320 | 3'390 | < quant. | 384'645 | 8'720 | 1'190 | < quant. |  |
| 29 | 17'390 | 940 | < quant. | 418'700 | 7'710 | 1'020 | < quant. |  |
| 33 | 11'370 | 4'970 | < quant. | 289'400 | 21'810 | 1'420 | < quant. |  |
| 36 | 24'820 | 7'360 | < quant. | 618'055 | 8'310 | 890 | < quant. |  |
| 40 | 6'160 | 9'260 | < quant. | 454'360 | 9'520 | < quant. | < quant. |  |
| 43 | 89'910 | 5'860 | 760 | 424'600 | 101'570 | 3'260 | 2'160 |  |
| 47 | 7'710 | 6'470 | < quant. | 269'200 | 3'290 | 1'120 | 1'640 |  |
| 50 | 194'060 | 208'240 | < quant. | 803'900 | 462'370 | 16'640 | < quant. |  |
| 54 | 50'100 | 238'700 | < quant. | 412'000 | 372'550 | < quant. | < quant. |  |
| 57 | 10'140 | 14'890 | < quant. | 271'640 | 73'740 | < quant. | 1'040 |  |
| 64 | 21'370 | 10'860 | < quant. | 271'700 | 18'640 | 940 | < quant. |  |
| 68 | 5'020 | 2'640 | < quant. | 289'285 | 8'140 | < quant. | < quant. |  |
| 71 | 8'610 | 1'470 | < quant. | 285'490 | 9'110 | < quant. | < quant. |  |
| average | 23'962 |  |  | 410'940 | 56'694 |  |  |  |
| st. dev. | 45'150 |  |  | 132'993 | 126'792 |  |  |  |
| median | 7'515 | 4'970 | < quant. | 415'350 | 8'225 | < quant. | < quant. |  |

Table S.3. Total cell counts (TCC) and intact cell counts (ICC) from the effluent of the GAC filter. This data was collected after the conclusion of other monitoring in this study. st. dev. indicates standard deviation.

| Time (hr) | Total cell count (#/ml) | Intact cell count (#/ml) |  |
| --- | --- | --- | --- |
| 0 | 312'333 | 183'000 |  |
| 12 | 208'667 | 165'333 |  |
| 24 | 279'333 | 156'667 |  |
| 36 | 346'000 | 191'333 |  |
| 48 | 306'000 | 177'000 |  |
| 60 | 372'000 | 186'667 |  |
| 72 | 306'667 | 152'333 |  |
| 84 | 365'000 | 180'000 |  |
| 96 | 286'667 | 177'667 |  |
| average | 309'185 | 174'444 |  |
| st. dev. | 49'931 | 13'433 |  |
| median | 306'667 | 177'667 |  |
